# Identification and comprehensive analysis of gene characteristics related to chromatin remodeling in thyroid cancer patients

**DOI:** 10.1097/MD.0000000000048824

**Published:** 2026-05-15

**Authors:** Shigui Wang, Shuangshuang Sun, Xiangzhong Wang, Xiang Chen

**Affiliations:** a Department of General Surgery, Jurong Hospital Affiliated to Jiangsu University, Jurong City, Jiangsu, China; b Department of Oncology, Jurong Hospital Affiliated to Jiangsu University, Jurong City, Jiangsu, China.

**Keywords:** bioinformatics, biomarkers, chromatin remodeling, thyroid cancer

## Abstract

Thyroid cancer (THCA) is the 9th most common endocrine tumor worldwide. However, its etiology and pathogenesis are not fully understood. Therefore, this study aimed to identify the biomarkers associated with chromatin remodeling in patients with THCA. THCA-related datasets and chromatin remodeling related genes were included in this study. Differential expression analysis and 2 machine learning algorithms were employed to identify candidate genes. Biomarkers were identified by receiver operating characteristic curve analysis. The prognostic potential of the biomarkers was explored using Kaplan–Meier (KM) survival analysis. Key genes linked to biomarkers were identified using weighted gene co-expression network analysis. Immune infiltration analysis was performed to explore differences in immune infiltration between THCA and control groups. Finally, the expression for biomarker was validated in clinical samples using reverse transcription quantitative polymerase chain reaction. Five biomarkers (CHD4, SMARCA2, CHD3, ATAD2, and SMARCA4) were screened. KM survival analysis revealed that patients with higher expression of SMARCA4, CHD4, and ATAD2 had a higher survival rate, whereas in the lower expression groups of CHD3 and SMARCA2, the survival rate of THCA patients was lower. A total of 98 genes related to biomarkers were identified using weighted gene co-expression network analysis. In addition, a total of 20 immune cells infiltrated differentially in THCA and controls, with the largest positive correlation between immature dendritic cells and ATAD2, with a correlation coefficient of 0.54, and a large positive correlation between CD56dim natural killer cells and SMARCA4, which was 0.5. Reverse transcription quantitative polymerase chain reaction revealed that the expression of biomarkers was consistent with the results of the bioinformatics analysis. In summary, SMARCA4, CHD4, and ATAD2 were overexpressed, whereas CHD3 and SMARCA2 were downregulated in THCA samples. This study identified 5 biomarkers (CHD4, SMARCA2, CHD3, ATAD2, and SMARCA4) associated with chromatin remodeling in THCA. Current reference points for the prevention and treatment.

## 1. Introduction

Thyroid cancer (THCA) is the most common endocrine tumor and the 9th most common cancer worldwide. The incidence of common tumors has steadily increased over the past few decades.^[1]^ With the incidence of THCA, its treatment of THCA is becoming increasingly standardized. Although most cases of early-stage thyroid carcinoma (THCA) can be cured by conventional treatments, including surgical resection, radioactive iodine therapy, thyroid stimulating hormone -suppressive thyroxine therapy, chemotherapy, and radiotherapy,^[2]^ the prognosis of patients with locally advanced or metastatic disease remains unsatisfactory. This is mainly due to the fact that THCA cells often develop resistance to multiple therapeutic approaches, such as radiotherapy, chemotherapy, multi-kinase inhibitors, and immune checkpoint inhibitors.^[3]^ These add challenges to subsequent treatment and prognosis rehabilitation management. Therefore, it is necessary to study the diagnostic markers and potential therapeutic targets of THCA to further understand the functional mechanisms underlying its development.

The remodeling of chromatin alters the transcription mechanism of DNA. Recent studies have shown that chromatin is highly dynamic and its filamentous structure is frequently modified by various complexes. The chromatin structure affects many aspects of DNA replication, recombination, repair, and transcriptional control.^[4]^ In addition, widely developed molecular biological techniques have led to a new understanding of the role of chromatin remodeling in the development of diseases, especially cancers.^[5]^ A recent study found that the SWItch/sucrose nonfermentable (SWI/SNF) chromatin remodeling complex is key to maintaining THCA differentiation, and its loss leads to intolerance to radioactive iodine and resistance to mitogen-activated protein kinase inhibitor-based redifferentiation therapies.^[6]^ Thyroid receptor beta (TRβ)-mediated gene inhibition was found via brahma‐related gene 1 dependent chromatin remodeling.^[7]^ In addition, loss of TRβ function can lead to the growth of THCA. TRβ-mutant variant is known to exhibit resistance to thyroid hormones and promote the development of follicular THCA in a mouse model.^[8]^ These results suggest that chromatin remodeling has great potential value in tumor progression, prognosis, and clinical therapy. However, the role of chromatin remodeling mechanisms in THCA is not fully understood and requires further exploration.

To investigate the role of chromatin remodeling in THCA patients, differential analysis, enrichment analysis, and machine learning were conducted to obtain differential genes, functional pathways, interaction between genes, and key genes. Expression verification experiments, KM curve analysis, clinical characteristic analysis of biomarkers, weighted gene co-expression network analysis (WGCNA) analysis, and immune infiltration analysis were performed systematically and comprehensively. Moreover, elucidating the occurrence and development mechanisms of THCA at the molecular level and developing new biomarkers and therapeutic targets for the prevention and treatment of THCA are important.

## 2. Materials and methods

### 2.1. Data source

The THCA-related RNA-seq dataset was downloaded from The Cancer Genome Atlas (TCGA) database (http://www.cancer.gov). The TCGA-THCA database was downloaded and contained 552 samples (496 thyroid samples from the THCA group and 56 paracancerous tissue samples). The GSE33630 dataset was downloaded from the Gene Expression Omnibus database (https://www.ncbi.nlm.nih.gov/geo/). The dataset contained 60 papillary thyroid carcinoma (PTC) samples and 45 paraneoplastic tissue samples. Chromatin remodeling related genes (CRRGs) were obtained from published literature and merged. After de-emphasis, a total of 177 CRRGs were obtained.^[9]^

### 2.2. Identification and analysis of candidate genes

Differentially expressed genes (DEGs) were obtained by comparing the differences in gene expression between THCA samples and normal samples using the “DESeq 2” package (version 1.34.0) in the R language. (|log_2_ FC| > 0.5, *P* value <.05).^[10]^ Candidate genes were identified by overlapping the DEGs and CRRGs.^[10]^ To explore the biological functions and signaling pathways of candidate genes. Based on candidate genes, gene ontology enrichment of candidate genes, including biological process, cellular component, molecular function, and Kyoto encyclopedia of genes and genomes (KEGG), was performed using the R software package “clusterProfiler” (version 4.6.0). KEGG pathway enrichment was analyzed, and a *P* value of <.05 was considered as significantly enriched.^[11]^ Determine interactions between candidate genes. The resulting candidate genes were mapped to the Search Tool for the Retrieval of Interacting Genes/Proteins database (https://string-db.org/) online search tool to predict protein-functional associations and protein–protein interactions, with a confidence score >0.4. Visualized using Cytoscape software (Cytoscape Consortium, http://www.cytoscape.org/). The maximal clique centrality algorithm of the CytoHubba plug-in was used to score the genes selected from Search Tool for the Retrieval of Interacting Genes/Proteins database and the top 10 genes were selected as candidate key genes.^[12,13]^

### 2.3. Identification of biomarkers

For additional screening of biomarkers, based on the candidate key genes, further screening of the feature genes were performed by least absolute shrinkage and selection operator and support vector machine-recursive feature elimination (SVM-RFE) with the R package “glmnet” (version 4.1.4) and the R package “e1071” (version 1.7-13; Department of Statistics, TU Wien),^[11]^ respectively. Subsequently, the genes obtained from the 2 algorithms were intersected to obtain the feature genes using the “ggVennDiagram” software package (version 1.2.2). The receiver operating characteristic curves of the feature genes were plotted in GSE33630, and the genes with an area under the curve (AUC) >0.7 were selected as candidate biomarkers. Expression analysis was then performed, and the candidate biomarkers with discrepant expression levels between THCA and controls as well as consistent expression trends in TCGA-THCA and GSE33630 datasets were selected as biomarkers (*P* value <.05).^[14,15]^

### 2.4. Survival analysis and clinical profiling

To explore the prognostic potential of biomarkers, all THCA samples in TCGA-THCA were divided into high and low expression groups based on the optimal thresholds for biomarker expression, and Kaplan–Meier (KM) survival analysis was performed using the R package “survminer” to assess the survival differences of THCA patients in the high and low expression groups (*P* value <.05). To explore the correlation between biomarkers and clinical characteristics (age, sex, and pathologic_M). THCA samples containing clinical information from TCGA-THCA. Differences in biomarker expression between the groups were analyzed using the t-test or 1-way ANOVA (*P* value <.05).^[15]^

### 2.5. Weighted gene co-expression network analysis (WGCNA)

In this study, WGCNA was performed using the R package “WGCNA” (version 1.71; University of California)^[16]^ to screen for genes associated with biomarkers. First, the samples were clustered to identify and remove outliers. Next, a soft threshold was chosen to ensure the best agreement between gene interactions and the scale-free distribution. Subsequently, gene similarities were calculated using the neighbor-joining method to create a systematic clustering tree. A hybrid dynamic tree-cutting algorithm was used to create co-expression networks with each gene module containing at least 100 genes. Finally, the obtained modules were correlated with traits to identify key modules, and the genes in these key modules were designated as genes associated with biomarkers.^[16]^

### 2.6. Enrichment analyses of key genes

Key genes were identified by intersecting genes associated with biomarkers and DEGs. They were subjected to gene ontology and KEGG enrichment analyses to explore their functions (*P* value <.05).

### 2.7. Immune infiltration analysis

To obtain a comprehensive picture of the infiltration landscape of immune cells in the THCA and normal groups. The abundance values of immune cells in all samples of the TCGA-THCA dataset were calculated using ssGSEA (Broad Institute, Inc., and University of California), followed by a *t* test to analyze the differences between immune cells in THCA and normal controls.^[17]^ To understand the correlation between biomarkers and immune cells, Spearman correlation analysis of biomarkers and differentiated immune cells was performed based on all samples in the training set.^[18]^

### 2.8. Experimental verification

This study was approved in accordance with the Ethical Standards of the Institutional Ethics Committee of Jurong Hospital Affiliated to Jiangsu University, and the ethics approval number is JRH-IEC-2024018. All patients written informed consent was obtained. After obtaining approval from the Ethics Committee of our hospital, we extracted 5 pairs of tissue samples (cancerous tissue and adjacent tissue) from patients who were clearly diagnosed with THCA and underwent concurrent surgery. The total RNA extraction kit, reverse transcription kit and 2× Universal Blue SYBR Green qPCR Master Mix were purchased from Wuhan Xavier Biotechnology Co., Ltd., and the target gene primer sequence was synthesized by Wuhan Xavier Biotechnology Co., Ltd.. The internal reference gene GAPDH sequence: justice chain is 5′-GGAAGCTTGTCATCAATGGAAATC-3′, antisense chain is 5′-TGATGACCCTTTTGGCTCCC-3′; CHD4 sequence: justice chain is 5′-GGAAGCTTGTCATCAATGGAAATC-3′, antisense chain is 5′-TTTGGGCTCTGTCTCCATAGGT-3′; SMARCA2 sequence: justice chain is 5′-GTGTCTCCCATCCTATGCCGA-3′, antisense chain is 5′-ACATAGGGCTGGAGACGTGCT-3′; CHD3 sequence: justice chain is 5′-GAAAGCTGAAGGAGCAAGGACA-3′, antisense chain is 5′-CAGGAGGAAGCAGAATTGTTGG-3′; ATAD2 sequence: justice chain is 5′-AATGTGGAAATAACGGAGCAAC-3′, and antisense chain 5′-CCTCAATGACCGAGTAACTGGAA-3′. SMARCA4 sequence: the justice chain was 5′-CCGAGCAACCAACCACAAA-3′ and the antisense chain was 5′-GTTCCATCAAGCCTGAGGTATTT-3′.

All experimental steps for total RNA extraction were performed according to the manufacturer instructions. One microliter of the extracted RNA was used for concentration detection with a NanoPhotometer N50 (Implen GmbH, Munich, Germany), and the recorded purity/concentration was used to calculate the amount of RNA required for subsequent reverse transcription steps. Subsequently, the RNA was reverse transcribed into cDNA using Servicebio SweScript First Strand cDNA Synthesis Kit in accordance with the manufacturer instructions. Then, the cDNA was diluted 5 to 20 times with deionized water (without RNase/ARase), 3 μL of cDNA, 5 μL of 2× Universal Blue SYBR Green qPCR Master Mix, 1 μL of the forward primer (10 μM), and 1 μL of the reverse primer (10 μμM). Furthermore, 40 reaction cycles were conducted using a CFX96 real-time quantitative polymerase chain reaction instrument. The 2^−ΔΔCT^ method was used: A = cycle threshold (CT) (target gene, sample to be tested) − CT (internal standard gene, sample to be tested), B = CT (target gene, control sample) − CT (internal standard gene, control sample), K = A–B, the expression multiple = 2 − K was, and calculated the mean and standard deviation were calculated. SPSS software (IBM Corporation, IBM SPSS Statistics) was used to analyze the data, and the mean and standard deviation were calculated. A *t* test was carried out, and the difference was considered statistically significant if *p*- *P* value was <.05. Cancer tissue samples were taken as the experimental group, paracancer tissue as the control group, and expression multiple as the *Y*-axis variable. GraphPad Prism software (GraphPad Software, Inc., San Diego) was used to generate histograms showing the differences in desired gene expression.

### 2.9. Statistical analysis

The *t* test was used to compare differences between the 2 groups. ssGSEA-calculated abundance values. Spearman correlation coefficient was calculated using Spearman correlation coefficient. KM survival analysis with log-rank test was performed to assess significant differences in overall survival between the 2 groups. Statistical significance was set.

At *P* value <.05.^[19]^

## 3. Results

### 3.1. Totally 10 candidate key genes were identified

**Figure 1.**
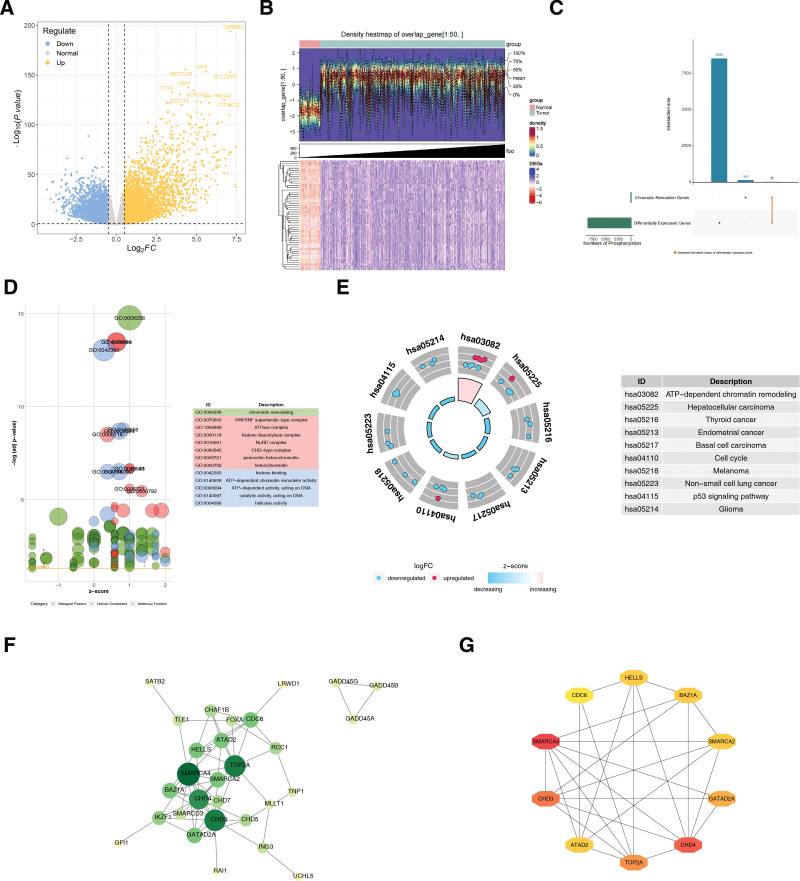
(A) Volcano map of differentially expressed genes. Each dot in the figure represents a gene, with yellow representing upregulated differential genes, blue representing downregulated differential genes, and gray representing nonsignificant differential genes. (B) Heat map of differentially expressed genes. Each column represents a sample, each row represents the expression level of each gene in a different sample, and the heatmap color represents the amount of gene expression in the sample. Green indicates normal samples and red indicates diseased samples. (C) The intersection of differentially expressed genes and 177 chromatin remodelling complex-associated genes. (D) GO enrichment analysis of candidate genes. Circles in the figure represent enriched pathways, with different colors denoting their classification under cellular component, molecular function, and biological process. The table on the right lists the enriched pathways. (E) Intersection gene from KEGG enrichment analysis. The blue represents downregulated genes, red represents upregulated genes, and the table on the right represents enriched pathways. (F) Intersection gene from PPI network. Each node represents an intersection gene and the lines represent interactions between genes. (G) Candidate key gene PPI network. Each node represents each gene, and the lines represent the interactions between genes. GO = gene ontology, KEGG = Kyoto encyclopedia of genes and genomes, PPI = protein–protein interaction.

A total of 8591 DEGs were identified through differential expression analyses, of which 4257 were upregulated and 4334 were downregulated (Fig. 1A and B). A total of 36 candidate genes were obtained by taking the intersection of 8591 DEGs and 177 genes associated with chromatin remodeling complexes (Fig. 1C). These candidate genes were enriched in chromatin remodeling, the SWI/SNF superfamily type complex, ATPase complex, histidine deacetylase complex, and nucleosome remodeling and deacetylase complex. Deacetylase complex and nucleosome remodeling and deacetylase complex (Fig. 1D). Candidate genes were significantly enriched in the adenosine triphosphate-dependent chromatin remodeling, hepatocellular carcinoma, THCA, endometrial cancer, and basal cell carcinoma pathways (Fig. 1E). The protein–protein interactions regulatory network of candidate genes contained 29 nodes and 62 interaction pairs, including SMARCA4-CHD4 and CHD3-TOP2A (Fig. 1F). Using the maximal clique centrality algorithm of the CytoHubba plugin, the top 10 genes were screened as candidate key genes: SMARCA4, CHD4, CHD3, TOP2A, GATAD2A, BAZ1A, SMARCA2, and ATAD2. HELLS and CDC6 (Fig. 1G).

### 3.2. CHD4, SMARCA2, CHD3, ATAD2 and SMARCA4 served as biomarkers in THCA

**Figure 2.**
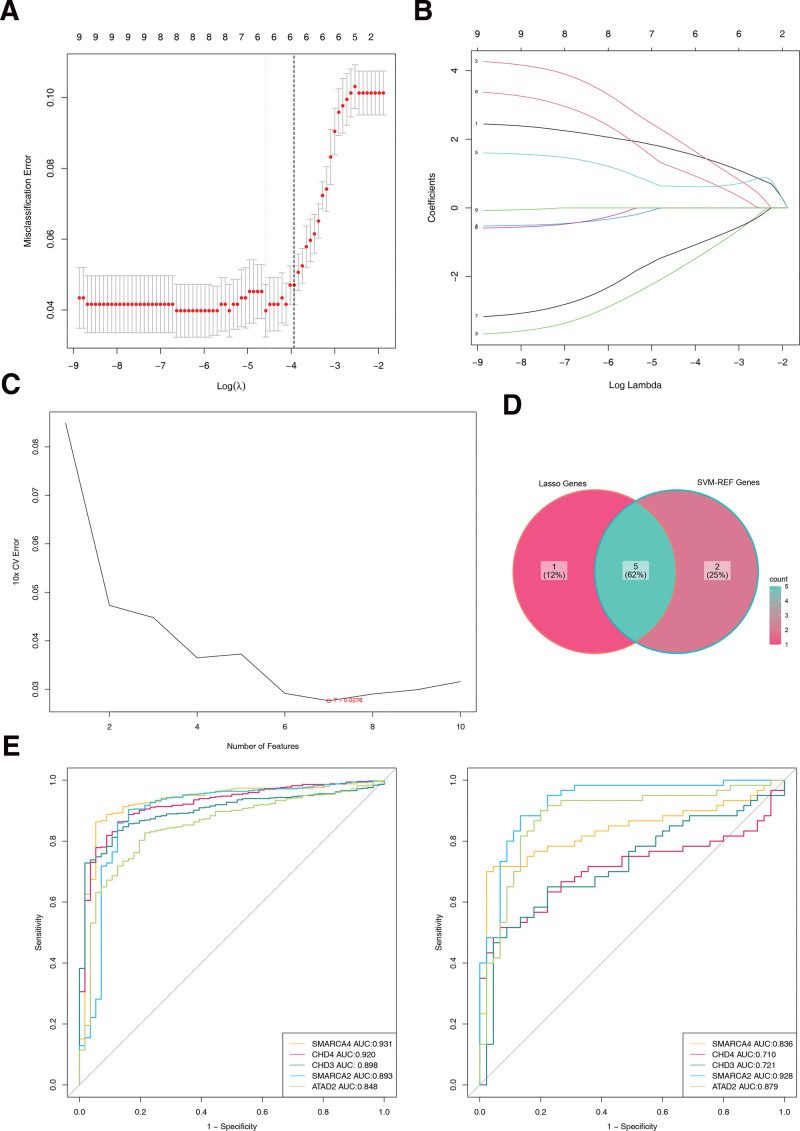
Genes from LASSO machine learning algorithms. (A) Each curve represents the change trajectory of each independent variable coefficient, the ordinate is the coefficient value, and the abscissa is the model at this time which the number of nonzero coefficients. (B) The abscissa is log(Lambda), and the ordinate represents the error of cross-validation. The red dots represent the mean square error and 1 standard deviation up or down. The smaller the mean square error, the better the model; The number above indicates the number of independent variables still present in the model (not necessarily monotonically decreasing). The 1st dotted line indicates the minimum mean square error; The 2nd dotted line shows the position of 1 standard deviation at the lowest point, representing the simplest model that can be obtained with 1 standard deviation sacrificed. (C) SVM-RFE result. The horizontal coordinate represents the number of features, and the vertical coordinate represents the error rate of the curve changes after a 10-fold cross-validation. The circles represent the lowest error rate for curve changes after 10× cross-validation. (D) Venn map of characteristic genes. The pink module on the left represents genes screened by Lasso, and the gray pink module on the right represents genes screened by SVM-RFE. (E) ROC curve analysis and verification of characteristic genes. (left: training tests; right: validation tests). LASSO = least absolute shrinkage and selection operator, SVM-RFE = support vector machine-recursive feature elimination.

Five least absolute shrinkage and selection operator-featured genes (SMARCA4, CHD4, CHD3, GATAD2A, and SMARCA2) intersected with 7 SVM-REF-featured genes (CHD4, SMARCA2, CHD3, ATAD2, TOP2A, SMARCA4, and BAZ1A), producing 5 feature genes (CHD4, SMARCA2, CHD3, ATAD2, and SMARCA4) (Fig. 2A–D). Receiver operating characteristic curves showed that the AUCs of the 5 feature genes were all >0.7 in both TCGA-THCA and GSE33630 datasets. Consequently, they were used as biomarkers for subsequent analyses (Fig. 2E).

### 3.3. The expression of CHD4, SMARCA2, CHD3, ATAD2 and SMARCA4 in THCA and normal samples

**Figure 3.**
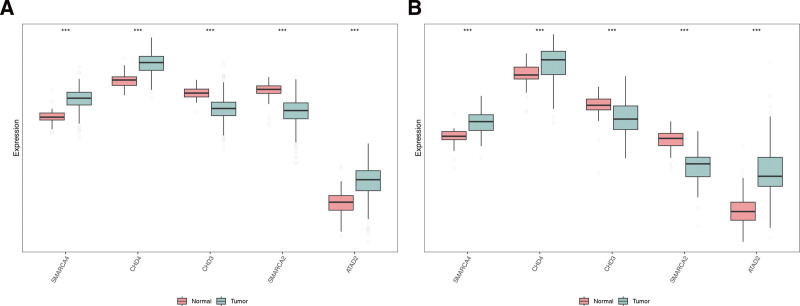
Biomarker differential expression changes green is THCA sample, red is normal sample. (A) The expression of biomarkers in the training set. (B) The expression of biomarkers in the verification set. THCA = thyroid cancer.

The results showed that in the training set, 5 biomarkers were significantly different between the THCA and normal samples, and the expression of SMARCA4, CHD4, and ATAD2 was significantly higher in the THCA samples, but the expression of CHD3 and SMARCA2 was significantly lower. In addition, in the validation set, the 5 biomarkers were significantly different between the THCA and normal samples, and the trend was consistent with the training set (Fig. 3A and B).

### 3.4. High expression of SMARCA4, CHD4 and ATAD2 might increase the survival probability of THCA patients

**Figure 4.**
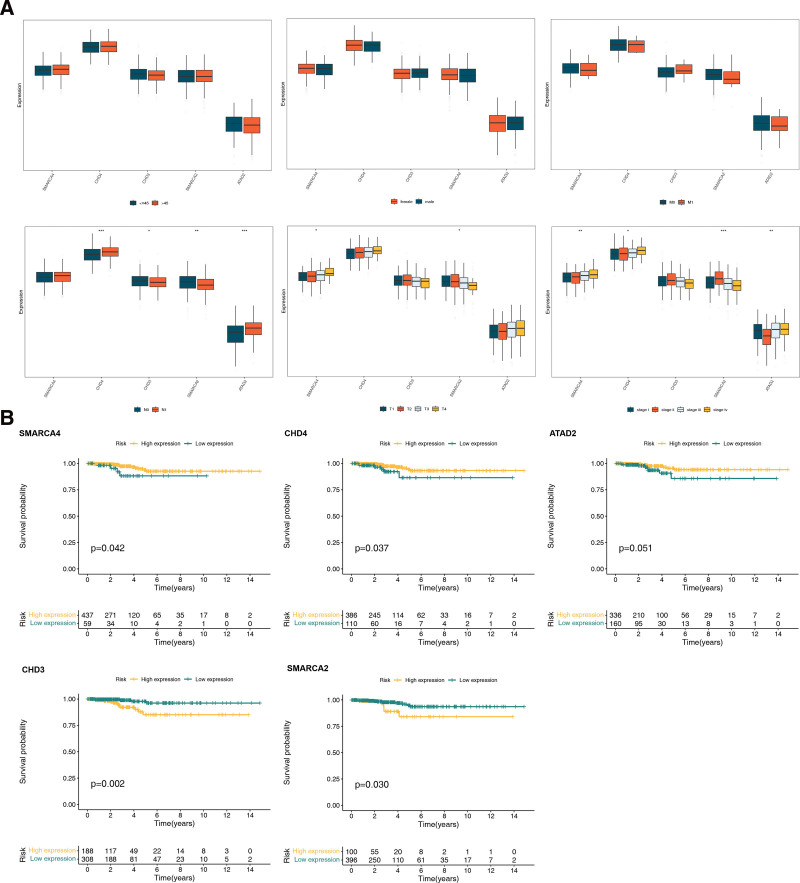
(A) Analysis of clinical characteristics of biomarkers. Different colors in the diagram represent different groups. (B) KM survival analysis of biomarkers. The high expression group is shown in yellow and the low expression group is shown in green. KM = Kaplan–Meier.

We found no significant expression of biomarkers when age, sex, and pathologic_M were used as clinical features. However, significant differences were observed in the expression of CHD4, CHD3, SMARCA2, and ATAD2 when pathologic_N was used as a clinical feature, and significant differences were observed in the expression of SMARCA4 and SMARCA2 when pathologic_T and stage were used as clinical features (Fig. 4A). KM survival analysis revealed that patients with higher expression of SMARCA4, CHD4, and ATAD2 had a higher survival rate, whereas in the lower expression groups of CHD3 and SMARCA2, the survival rate of THCA patients was lower (Fig. 4B).

### 3.5. WGCNA acquisition of genes associated with biomarkers

**Figure 5.**
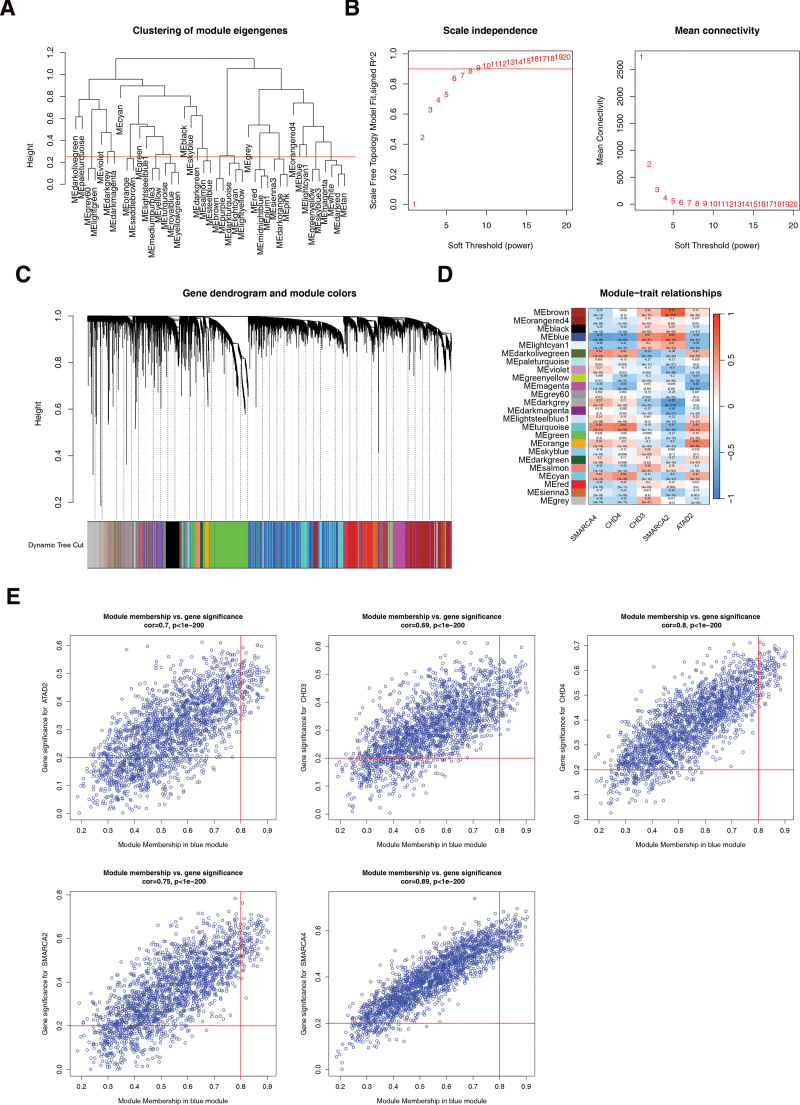
(A) Clustering of module eigengenes. The branch represents the sample and the ordinate represents the height of the hierarchical cluster. The red line represents the outlier samples. (B) The selection of soft threshold β. The horizontal axis represents the power value of the weight parameter, the vertical axis of the left figure is the scale-free fit index (signed *R*^2^), and the vertical axis of the right figure represents the mean value of all the gene adjacency functions in the corresponding gene module. (C) Module cluster diagram. The upper part is the hierarchical clustering tree of genes, and the lower part is the gene module. The genes that cluster into the same branch are divided into the same module, with different colors representing different modules. (D) Heat map of the relationship between traits, gene modules and traits based on biomarkers. The darker the color, the higher the correlation, with red being positively correlated and blue being negatively correlated. The numbers in the cell represent the correlation and significance, the top row is the correlation, the bottom row is the *P* value (significance), the left side is the gene module of different colors, and the color bar on the right represents the correlation range. (E) Key module gene screening. The horizontal and vertical lines represent GS and MM thresholds, respectively. GS = gene significance, MM = module membership.

First, the samples were clustered and analyzed using the samples below the red line (Fig. 5A). The soft threshold was determined to be 8 when *R*^2^ exceeded 0.85, and the mean connectivity approached zero (Fig. 5B). A total of 23 modules were segmented with a minimum number of genes per gene module of 25, a total of 23 modules were segmented and gray modules were excluded (Fig. 5C). Finally, 98 genes associated with biomarkers were identified based on the 1989 genes screened in the BLUE module (Fig. 5D and E).

### 3.6. Acquisition and analysis of key genes

**Figure 6.**
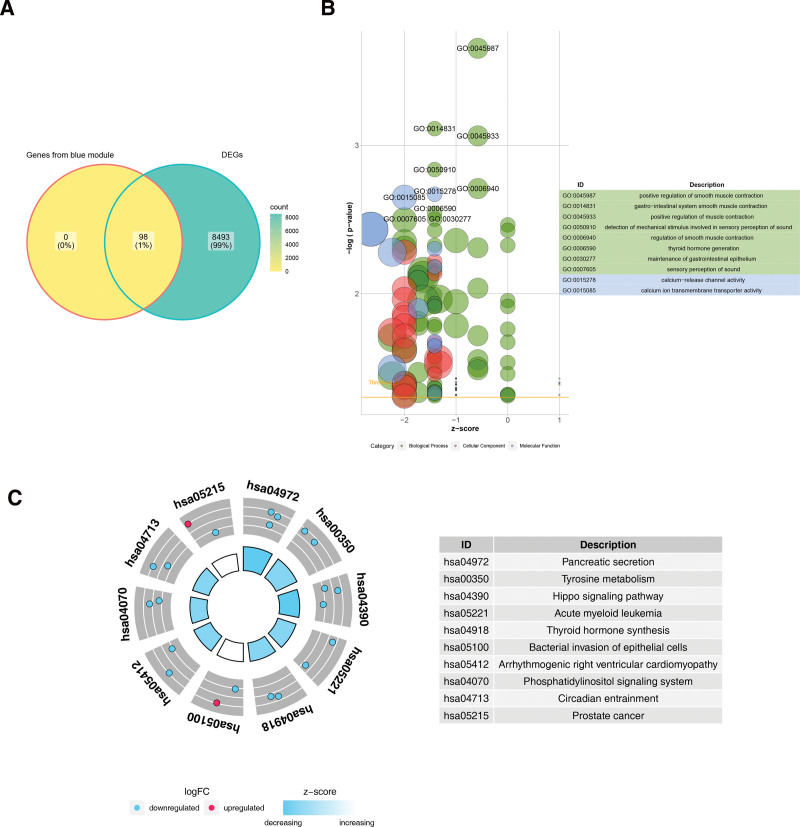
(A) Venn map of key genes. The yellow module on the left represents the genes screened by the key module and the green module on the right represents the differential genes. (B) GO enrichment analysis of key genes. The circles in the figure represent different enriched pathways, the different colors indicate that these pathways belong to CC, MF, and BP, and the table on the right represents the top ten enriched pathways. (C) KEGG enrichment analysis of key genes. The blue and red dots represent different genes, with blue representing downregulated genes, red representing upregulated genes, and the table on the right represents the top ten enriched pathways. BP = biological process, CC = cellular component, GO = gene ontology, KEGG = Kyoto encyclopedia of genes and genomes, MF = molecular function.

The 98 genes associated with biomarkers and the 8591 DEGs were crossed, obtaining a total of 98 key genes were identified (Fig. 6A). Key genes were found to be enriched in the positive regulation of smooth muscle contraction, smooth muscle contractions in the gastrointestinal system, positive regulation of smooth muscle contractions, detection of mechanical stimuli in perception, and positive regulation of muscle contractions (Fig. 6B). In addition, they were significantly enriched in pathways for the detection of mechanical stimuli in sound perception and regulation of smooth muscle contractions (Fig. 6C).

### 3.7. Totally 20 immune cells were infiltrated differentially in THCA and controls

**Figure 7.**
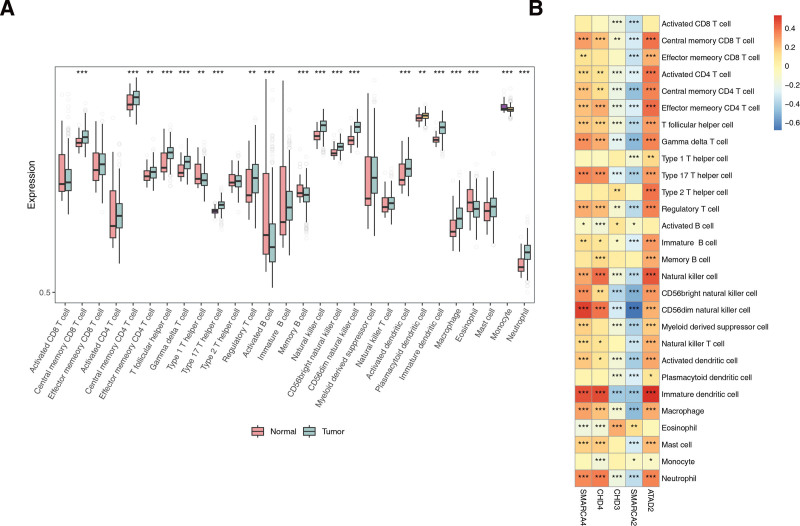
(A) Differences in the abundance of immune cells between THCA and normal controls. (B) Heatmap showing the correlation between biomarkers and differentially expressed immune cells. THCA = thyroid cancer.

A total of 20 immune cells differed between the THCA and normal groups, including activated cluster of differentiation 8+ T cells and central memory cluster of differentiation 8+ T cells (Fig. 7A). The results showed a strong positive correlation between immature dendritic cells and ATAD2, with a correlation coefficient of 0.54, and a strong positive correlation between CD56dim natural killer cells and SMARCA4, with a correlation coefficient of 0.5. The strongest negative correlation was observed between CD56dim natural killer cells and SMARCA2, with a correlation coefficient of −0.68 (Fig. 7B).

### 3.8. The expression trend of biomarkers in clinical samples was consistent with the results of bioinformatics

**Figure 8.**
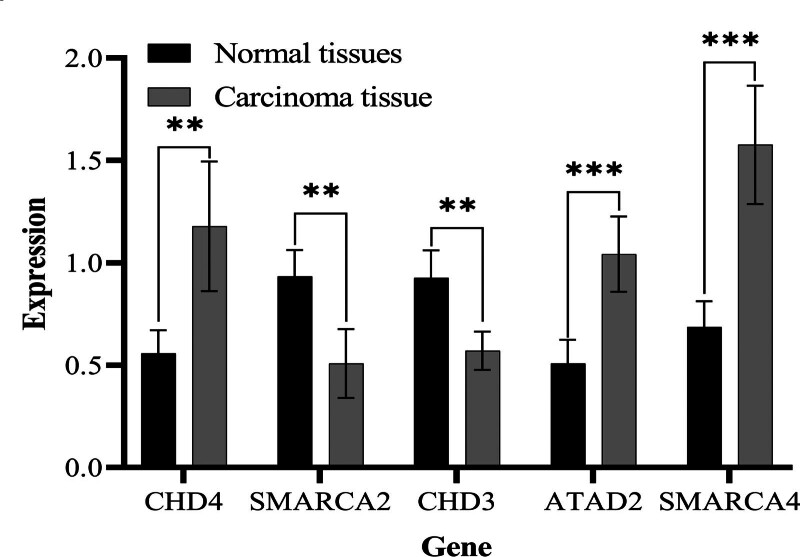
The expression multiples of 5 genes were compared by histogram.

According to the statistical data, the expression levels of SMARCA4, CHD4, and ATAD2 in THCA tissues were significantly higher than those in the paracancer tissue group, while the expression levels of CHD3 and SMARCA2 in THCA tissue were relatively lower than those in paracancer tissue (Table 1), with a *P* value <.05, considered statistically significant using SPSS software. The expression multiples of the 5 target genes in cancer tissues and adjacent tissues were compared by histogram (Fig. 8) using GraphPad Prism software.

**Table 1:** Statistical analysis of the expression levels of target genes by SPSS, a *P* value was considered statistically significant.

	Control	THCA	*P* value
CHD4	0.558 ± 0.112	1.178 + 0.317	.003
SMARCA2	0.934 ± 0.129	0.508 + 0.168	.002
CHD3	0.926 ± 0.135	0.57士0.093	.0013
ATAD2	0.508 ± 0.115	1.042士0.184	.006
SMARCA4	0.686 ± 0.126	1.576士0.288	.0002

THCA = thyroid cancer.

## 4. Discussion

The incidence of THCA has increased significantly worldwide and is growing faster than that of other tumors. Some special pathological types of THCA are characterized by high rates of aggression and distant metastasis and are found at late stages, leading to a significant decline in survival.^[20–22]^ Chromatin remodeling is a series of biological processes mediated by chromatin remodeling complexes that are fundamentally characterized by changes in nucleosomes on chromatin.^[23]^ Nucleosomes play a crucial rule in controlling gene expression, but a complex cellular mechanism working in concert with transcription factors is required to mobilize nucleosomes to control gene expression.^[24]^ Chromatin remodeling occurs in many cancers and is considered a potential marker for cancer development and progression.^[25]^ Chromatin remodeling is considered a contributing factor in the occurrence and progression of THCA. TRβ induces local nucleosomal structural recombination, resulting in changes in chromatin accessibility, which leads to the expression or inhibition of some genes and changes in related expression pathways, resulting in the occurrence and development of disease.^[7]^ Therefore, it is urgent to search for biomarkers related to chromatin and THCA to aid in the diagnosis and treatment of this disease.

The chromosomal structural domain-helicase DNA-binding proteins (CHD) family consists of proteins involved in the epigenetic regulation of chromatin remodeling, which use the energy generated by adenosine triphosphate hydrolysis to remodel chromatin structure and regulate the attachment of transcription factors to genes. CHDs are involved in cellular transcription, proliferation, and DNA damage repair, as well as in the regulation of stem cell survival.^[26–29]^ Of them, CHD3 acts through covalent modification with histone H3K9^[30]^ and is involved in DNA damage repair.^[31]^ Frameshift mutations and loss of CHD3 expression are common in gastric and colorectal cancers with microsatellite instability‐high. These changes may contribute to cancer pathogenesis by unwinding the CHD-mediated regulation of chromatin remodeling regulation.^[32]^ CHD3 and CHD4 were inextricably linked in the study by Helen Hoffmeister et al, CHD3 levels in thymus of mice were elevated after CHD4 was knocked out.^[33]^

CHD4 is located in human chromatin 12p13 and is involved in chromatin reorganization and DNA damage repair through the deacetylation of histones.^[34–36]^ Mutations in CHD4 are expressed in several cancer types. Le Gallo et al found somatic mutations in CHD4, which is a chromatin remodeling mutated gene, in 17% of patients with plasma cell endometrial cancer.^[37]^ Similarly, Zhao et al identified CHD4 mutations in 11 (21%) of 52 patients with uterine serous carcinoma.^[38]^ Kim et al reported that loss of CHD4 expression was observed in 56.4% of gastric cancers and 55.7% of colorectal cancers.^[32]^ CHD4 promotes cell proliferation, spheroid growth, migration, and progression of epithelial transition in PTC cells, and might be a promising target in the treatment of PTC, which overexpression in PTC patients can lead to poorer prognosis and poorer clinical staging.^[39]^

SWI/SNF chromatin remodeling complex plays an important role in chromatin remodeling and is composed of many variable subunits. It mainly includes 2 catalytic subunits (SMARCA2 and SMARCA4), 3 core subunits, and auxiliary regulatory subunits.^[40]^ SMARCA2 is critical for cellular metabolism, DNA repair, tumor angiogenesis, progression, and metastasis.^[41]^ SMARCA2 deficiency is an independent risk factor for the prognosis of gastric cancers by constructing a nomogram prognostic model^[42]^ and NSCLC patients in which the SMARCA2-negative group had a worse survival and prognosis than the SMARCA2-positive group.^[43]^ SMARCA4 is pivotal in gene transcription, differentiation, and DNA damage repair, which are similar to SMARCA1 and show high-frequency mutations in tumors.^[44]^ High expression of this gene is associated with poor prognosis in many types of tumors, including hepatocellular carcinoma and clear cell carcinoma of the kidney. The loss of SMARCA2 was discovered through genomic and transcriptomic landscapes in patients with anaplastic THCAs,^[45]^ which is consistent with our research.

ADAT2, also known as ANCCA, is located on human chromosome 8q24. The encoded protein contained 2 AAA regions and 1 bromine domain. ATAD2, a cofactor of c-myelocytomatosis oncogene, estrogen receptor α, andandrogen receptor, is regulated by androgens, estrogens, and E2Fs (E2F1–E2F3) and is significantly overexpressed in liver, prostate, lung, and ovarian cancers. High ATAD2 expression is associated with tumor stage, tissue grade, lymph node metastasis, and poor prognosis.^[46–48]^ A study by Sun et al showed that ATAD2 was significantly highly expressed in PTC tissues and correlated with tumor size. Downregulation of ATAD2 expression might significantly induce apoptosis of PTC cells and inhibit migration and invasion of PTC cells. NEAT1_2 plays a role in antiapoptosis and promotes PTC cell migration and invasion by regulating ATAD2 expression.^[49]^ The results of the above study are consistent with our research, which verifies the role of ATAD2 in the occurrence and development of THCA, and high expression of ATAD2 in THCA tissues. However, there are no relevant reports on the gene expression of SMARCA4 and CHD3 in THCA patients; therefore, this research has a certain innovative significance.

In summary, we identified, for the 1st time, a series of biomarkers associated with chromatin remodeling in THCA through TCGA and Gene Expression Omnibus databases and performed rigorous experimental validation. Five biomarkers were finally obtained with AUCs >0.7 on both the training and validation sets. Our study showed that immune cell infiltration is involved in the development of THCA. The study revealed significant positive correlations between some biomarkers and some immune cells (immature dendritic cells and ATAD2; CD56dim natural killer cells and SMARCA4) or negative correlations (CD56dim natural killer cells and SMARCA2), which may be involved in regulating the immune biological processes of epidemic cells. However, this study has some limitations. A more detailed genetic analysis of tissue samples from patients was lacking. However, the application of targeted drugs requires further clinical testing. Finally, the function of the desired gene needs to be studied further to explore its precise biological mechanism.

## 5. Conclusion

These 5 signature genes (SMARCA4, CHD4, ATAD2, CHD3, and SMARCA2) may be new potential biomarkers for the treatment of THCA patients, and high expression of SMARCA4, CHD4, and ATAD2 may increase the probability of survival in THCA patients. Conversely, low expression of CHD3 and SMARCA2 may reduce the probability of survival. These findings provide new insights into the diagnosis and treatment of TCHA.

## Author contributions

**Conceptualization:** Shuangshuang Sun, Shigui Wang

**Data curation:** Shigui Wang, Xiangzhong Wang

**Formal analysis:** Xiang Chen

**Investigation:** Shuangshuang Sun, Xiangzhong Wang

**Methodology:** Shuangshuang Sun, Xiangzhong Wang, Xiang Chen

**Project administration:** Shuangshuang Sun

**Resources:** Shigui Wang

**Software:** Shigui Wang, Xiang Chen

**Writing – original draft:** Shigui Wang

**Writing – review & editing:** Shuangshuang Sun, Xiangzhong Wang, Xiang Chen

## References

[1] Sung H Ferlay J Siegel RL. Global cancer statistics 2020: GLOBOCAN estimates of incidence and mortality worldwide for 36 cancers in 185 countries. CA Cancer J Clin. 2021;71:209–49. 33538338 10.3322/caac.21660

[2] Yang XY Yu Y Li DP Dong L. Current situation and thinking of diagnosis and treatment in some types of thyroid cancer. Zhonghua Er Bi Yan Hou Tou Jing Wai Ke Za Zhi. 2017;52:305–8. 28441814 10.3760/cma.j.issn.1673-0860.2017.04.017

[3] Catalano MG Fortunati N Boccuzzi G. Epigenetics modifications and therapeutic prospects in human thyroid cancer. Front Endocrinol (Lausanne). 2012; 3:40. 22649419 10.3389/fendo.2012.00040 PMC3355953

[4] Michael AK Thomä NH. Reading the chromatinized genome. Cell. 2021;184:3599–611. 34146479 10.1016/j.cell.2021.05.029

[5] Cheng ML Solit DB. Opportunities and challenges in genomic sequencing for precision cancer care. Ann Intern Med. 2018;168:221–2. 29310131 10.7326/M17-2940 PMC6659420

[6] Saqcena M Leandro-Garcia LJ Maag JLV. SWI/SNF complex mutations promote thyroid tumor progression and insensitivity to redifferentiation therapies. Cancer Discov. 2021;11:1158–75. 33318036 10.1158/2159-8290.CD-20-0735 PMC8102308

[7] Gillis NE Taber TH Bolf EL. Thyroid hormone receptor β suppression of RUNX2 is mediated by brahma-related gene 1-dependent chromatin remodeling. Endocrinology. 2018;159:2484–94. 29750276 10.1210/en.2018-00128 PMC6692870

[8] Lu C Zhu X Willingham MC Cheng SY. Activation of tumor cell proliferation by thyroid hormone in a mouse model of follicular thyroid carcinoma. Oncogene. 2012;31:2007–16. 21909131 10.1038/onc.2011.390 PMC3728834

[9] Zhang FL Li DQ. Targeting chromatin-remodeling factors in cancer cells: promising molecules in cancer therapy. Int J Mol Sci. 2022;23:12815. 36361605 10.3390/ijms232112815 PMC9655648

[10] Ritchie ME Phipson B Wu D. limma powers differential expression analyses for RNA-sequencing and microarray studies. Nucleic Acids Res. 2015;43:e47. 25605792 10.1093/nar/gkv007 PMC4402510

[11] Gong X Hou D Zhou S. FMO family may serve as novel marker and potential therapeutic target for the peritoneal metastasis in gastric cancer. Front Oncol. 2023;13:1144775. 37274237 10.3389/fonc.2023.1144775 PMC10234505

[12] Love MI Huber W Anders S. Moderated estimation of fold change and dispersion for RNA-seq data with DESeq2. Genome Biol. 2014;15:550. 25516281 10.1186/s13059-014-0550-8 PMC4302049

[13] Simon N Friedman J Hastie T Tibshirani R. Regularization paths for cox’s proportional hazards model via coordinate descent. J Stat Softw. 2011;39: 1–13. 10.18637/jss.v039.i05 PMC4824408 27065756

[14] Chen J Jin H Zhou H Hei X Liu K. Research into the characteristic molecules significantly affecting liver cancer immunotherapy. Front Immunol. 2023;14:1029427. 36860864 10.3389/fimmu.2023.1029427 PMC9968832

[15] Chen JW Dhahbi J. Lung adenocarcinoma and lung squamous cell carcinoma cancer classification, biomarker identification, and gene expression analysis using overlapping feature selection methods. Sci Rep. 2021;11:13323. 34172784 10.1038/s41598-021-92725-8 PMC8233431

[16] Tian Z He W Tang J. Identification of important modules and biomarkers in breast cancer based on WGCNA. Onco Targets Ther. 2020;13:6805–17. 32764968 10.2147/OTT.S258439 PMC7367932

[17] Zhou Y Tian Q Gao H. Immunity and extracellular matrix characteristics of breast cancer subtypes based on identification by T helper cells profiling. Front Immunol. 2022;13:859581. 35795662 10.3389/fimmu.2022.859581 PMC9251002

[18] Hu X Ni S Zhao K Qian J Duan Y. Bioinformatics-led discovery of osteoarthritis biomarkers and inflammatory infiltrates. Front Immunol. 2022;13:871008. 35734177 10.3389/fimmu.2022.871008 PMC9207185

[19] Bi KW Wei XG Qin XX Li B. BTK has potential to be a prognostic factor for lung adenocarcinoma and an indicator for tumor microenvironment remodeling: a study based on TCGA data mining. Front Oncol. 2020;10:424. 32351880 10.3389/fonc.2020.00424 PMC7175916

[20] Bray F Ferlay J Soerjomataram I Siegel RL Torre LA Jemal A. Global cancer statistics 2018: GLOBOCAN estimates of incidence and mortality worldwide for 36 cancers in 185 countries. CA Cancer J Clin. 2018;68:394–424. 30207593 10.3322/caac.21492

[21] Mao J Zhang Q Zhang H Zheng K Wang R Wang G. Risk factors for lymph node metastasis in papillary thyroid carcinoma: a systematic review and meta-analysis. Front Endocrinol (Lausanne). 2020;11:265. 32477264 10.3389/fendo.2020.00265 PMC7242632

[22] Dotinga M Vriens D van Velden FHP. Reinducing radioiodine-sensitivity in radioiodine-refractory thyroid cancer using lenvatinib (RESET): study protocol for a single-center, open label phase II trial. Diagnostics (Basel). 2021;11:2024. 36553163 10.3390/diagnostics12123154 PMC9777156

[23] Morrison AJ. Chromatin-remodeling links metabolic signaling to gene expression. Mol Metab. 2020;38:100973. 32251664 10.1016/j.molmet.2020.100973 PMC7300377

[24] Yaniv M. Chromatin remodeling: from transcription to cancer. Cancer Genet. 2014;207:352–7. 24825771 10.1016/j.cancergen.2014.03.006

[25] Nebbioso A Tambaro FP Dell’Aversana C Altucci L. Cancer epigenetics: moving forward. PLoS Genet. 2018;14:e1007362. 29879107 10.1371/journal.pgen.1007362 PMC5991666

[26] Hall JA Georgel PT. CHD proteins: a diverse family with strong ties. Biochem Cell Biol. 2007;85:463–76. 17713581 10.1139/O07-063

[27] Delmas V Stokes DG Perry RP. A mammalian DNA-binding protein that contains a chromodomain and an SNF2/SWI2-like helicase domain. Proc Natl Acad Sci U S A. 1993;90:2414–8. 8460153 10.1073/pnas.90.6.2414 PMC46097

[28] Micucci JA Sperry ED Martin DM. Chromodomain helicase DNA-binding proteins in stem cells and human developmental diseases. Stem Cells Dev. 2015;24:917–26. 25567374 10.1089/scd.2014.0544 PMC4390162

[29] Liu C Kang N Guo Y Gong P. Advances in chromodomain helicase DNA-binding (CHD) proteins regulating stem cell differentiation and human diseases. Front Cell Dev Biol. 2021;9:710203. 34616726 10.3389/fcell.2021.710203 PMC8488160

[30] Tencer AH Cox KL Di L. Covalent modifications of histone H3K9 promote binding of CHD3. Cell Rep. 2017;21:455–66. 29020631 10.1016/j.celrep.2017.09.054 PMC5653232

[31] Stanley FK Moore S Goodarzi AA. CHD chromatin remodeling enzymes and the DNA damage response. Mutat Res. 2013;750:31–44. 23954449 10.1016/j.mrfmmm.2013.07.008

[32] Kim MS Chung NG Kang MR Yoo NJ Lee SH. Genetic and expressional alterations of CHD genes in gastric and colorectal cancers. Histopathology. 2011;58:660–8. 21447119 10.1111/j.1365-2559.2011.03819.x

[33] Hoffmeister H Fuchs A Erdel F. CHD3 and CHD4 form distinct NuRD complexes with different yet overlapping functionality. Nucleic Acids Res. 2017;45:10534–54. 28977666 10.1093/nar/gkx711 PMC5737555

[34] Okada S Okazaki N. Chemotherapy in cancers of the gallbladder and bile ducts. Gan To Kagaku Ryoho. 1991;18:1269–72. 2069397

[35] Srinivasan R Mager GM Ward RM Mayer J Svaren J. NAB2 represses transcription by interacting with the CHD4 subunit of the nucleosome remodeling and deacetylase (NuRD) complex. J Biol Chem. 2006;281:15129–37. 16574654 10.1074/jbc.M600775200

[36] Ramírez J Dege C Kutateladze TG Hagman J. MBD2 and multiple domains of CHD4 are required for transcriptional repression by Mi-2/NuRD complexes. Mol Cell Biol. 2012;32:5078–88. 23071088 10.1128/MCB.00819-12 PMC3510529

[37] Le Gallo M O’Hara AJ Rudd ML. Exome sequencing of serous endometrial tumors identifies recurrent somatic mutations in chromatin-remodeling and ubiquitin ligase complex genes. Nat Genet. 2012;44:1310–5. 23104009 10.1038/ng.2455 PMC3515204

[38] Zhao S Choi M Overton JD. Landscape of somatic single-nucleotide and copy-number mutations in uterine serous carcinoma. Proc Natl Acad Sci USA. 2013;110:2916–21. 23359684 10.1073/pnas.1222577110 PMC3581983

[39] Pratheeshkumar P Siraj AK Divya SP. CHD4 predicts aggressiveness in PTC patients and promotes cancer stemness and EMT in PTC cells. Int J Mol Sci. 2021;22:504. 33419089 10.3390/ijms22020504 PMC7825451

[40] Narlikar GJ Sundaramoorthy R Owen-Hughes T. Mechanisms and functions of ATP-dependent chromatin-remodeling enzymes. Cell. 2013;154:490–503. 23911317 10.1016/j.cell.2013.07.011 PMC3781322

[41] Chetty R Serra S. SMARCA family of genes. J Clin Pathol. 2020;73:257–60. 32312722 10.1136/jclinpath-2020-206451

[42] Zhang Z Li Q Sun S. Expression of SMARCA2 and SMARCA4 in gastric adenocarcinoma and construction of a nomogram prognostic model. Int J Clin Oncol. 2023;28:1487–500. 37634210 10.1007/s10147-023-02403-0

[43] Sun S Li Q Zhang Z. SMARCA2 deficiency in NSCLC: a clinicopathologic and immunohistochemical analysis of a large series from a single institution. Environ Health Prev Med. 2022;27:3. 35289322 10.1265/ehpm.21-00254 PMC9093611

[44] Masliah-Planchon J Bièche I Guinebretière JM Bourdeaut F Delattre O. SWI/SNF chromatin remodeling and human malignancies. Annu Rev Pathol. 2015;10:145–71. 25387058 10.1146/annurev-pathol-012414-040445

[45] Kasaian K Wiseman SM Walker BA. The genomic and transcriptomic landscape of anaplastic thyroid cancer: implications for therapy. BMC Cancer. 2015;15:984. 26680454 10.1186/s12885-015-1955-9 PMC4683857

[46] Kiesel LA Rody A Greb RR Szilágyi A. Clinical use of GnRH analogues. Clin Endocrinol (Oxf). 2002;56:677–87. 12072036 10.1046/j.1365-2265.2002.01291.x

[47] Erzberger JP Berger JM. Evolutionary relationships and structural mechanisms of AAA+ proteins. Annu Rev Biophys Biomol Struct. 2006;35:93–114. 16689629 10.1146/annurev.biophys.35.040405.101933

[48] Wendler P Ciniawsky S Kock M Kube S. Structure and function of the AAA+ nucleotide binding pocket. Biochim Biophys Acta. 2012;1823:2–14. 21839118 10.1016/j.bbamcr.2011.06.014

[49] Sun W Lan X Zhang H. NEAT1_2 functions as a competing endogenous RNA to regulate ATAD2 expression by sponging microRNA-106b-5p in papillary thyroid cancer. Cell Death Dis. 2018;9:380. 29515109 10.1038/s41419-018-0418-z PMC5841310

